# Supplementary material for: Chromothripsis during telomere crisis is independent of NHEJ, and consistent with a replicative origin
Source: Genome Res. 2019 May;29(5):737–49. doi: 10.1101/gr.240705.118 (PMC6499312; doi:10.1101/gr.240705.118)
Supplement: Supplemental Material [file supp_gr.240705.118_Supplemental_file_1.zip › contigs/annotated_contigs/DB92/contig.2.DB92_length_687_mean_cov_5.02183406114.docx]

**DB92_length_687_mean_cov_5.02183406114**

```
CTTGAAATATAAGGGGGCAAGTTTAGAGAGATCAGATCCCAGGAAAGATAGAGCTTTTAAACTCTTCATTTCTCCTAAGAAAAGCACCA
 >chrX:97700924-97701181 - E=7e-143
TTCCCTACTAATCCTAATAAAGGGAGTTTGGAGTAATGGTGAAAAGTAGGGAACATTGATGCCAGACTGACTGGATTCAAATCGTGTTT

TTGACACTTACCGTATGTGTGTCTTTGGACAAAATACTTAAATTTCCTCTGCCTTAGTTTCTCTATCTTGAAATGGTGA|AATTTATTC
                                                                                 >chrX:1
ATAAATAAAATAATTGCTTATAGGAAAAATATTTCAATTGAAATTTCATTTTCCTCAAAACATTTACCAATTTTTAAGCTGTGTAAGTG
1309215-11309645 + E=5e-244
GGGCCTAAATTAGTGGAAGAGATTTCAAACAGAGATCCATATTTTAATGTCCTATGATTCAGGATATGCAGTAGGGGAAAATCAATTAT

TTCTATGGAATCGGCCATCAAAGCTTGTTCAACTGTCCACGGTGAATTAGCGGTAACTCACTATCCAGTGGTCCAGAGGAATTAATGCC

ACATTAAGGCAGCATCCTGTTGATCATTATGGCCAAATATATCATCCTCTCATTCTTAGAAGGTGAGAAAAAGTATAAGATCAATCTTT

CTAAAACTACAAGGCTACTAGTAATTCATCATTGACTTCATTAGTAGAGATTACCCACAACTTTG
```
